# Price elasticity of the demand for soft drinks, other sugar-sweetened beverages and energy dense food in Chile

**DOI:** 10.1186/s12889-017-4098-x

**Published:** 2017-02-10

**Authors:** Carlos M. Guerrero-López, Mishel Unar-Munguía, M. Arantxa Colchero

**Affiliations:** 1 Center for Health Systems Research, Instituto Nacional de Salud Pública, Universidad No. 655 Colonia Santa María Ahuacatitlán, Cerrada Los Pinos y Caminera, Cuernavaca, Morelos C.P. 62100 Mexico; 2 Nutrition and Health Research Center, Instituto Nacional de Salud Pública, Universidad No. 655 Colonia Santa María Ahuacatitlán, Cerrada Los Pinos y Caminera, Cuernavaca, Morelos C.P. 62100 Mexico

**Keywords:** Price elasticity, Soft drinks, Sugar sweetened beverages, High energy density foods, Chile

## Abstract

**Background:**

Chile is the second world’s largest per capita consumer of caloric beverages. Caloric beverages are associated with overweight, obesity and other chronic diseases. The objective of this study is to estimate the price elasticity of demand for soft drinks, other sugar-sweetened beverages and high-energy dense foods in urban areas in Chile in order to evaluate the potential response of households’ consumption to changes in prices.

**Methods:**

We used microdata from the VII Family Budget Survey 2012–2013, which collects information on expenditures made by Chilean urban households on items such as beverages and foods. We estimated a Linear Approximation of an Almost Ideal Demand System Model to derive own and cross price elasticities of milk, coffee, tea and other infusions, plain water, soft drinks, other flavored beverages, sweet snacks, sugar and honey, and desserts. We considered the censored nature of the data and included the Inverse Mills Ratio in each equation of the demand system. We estimated a Quadratic Almost Ideal Demand System and a two-part model as sensitivity analysis.

**Results:**

We found an own price-elasticity of −1.37 for soft drinks. This implies that a price increase of 10% is associated with a reduction in consumption of 13.7%. We found that the rest of food and beverages included in the demand system behave as substitutes for soft drinks. For instance, plain water showed a cross-price elasticity of 0.63: a 10% increase in price of soft drinks could lead to an increase of 6.3% of plain water. Own and cross price elasticities were similar between models.

**Conclusions:**

The demand of soft drinks is price sensitive among Chilean households. An incentive system such as subsidies to non-sweetened beverages and tax to soft drinks could lead to increases in the substitutions for other healthier beverages.

## Background

Overweight and obesity have increased in less developed countries at a noticeable pace [1]. It is estimated that overweight and obesity caused 3.4 million deaths and 93.6 million DALYs in 2010 [2]. Latin American countries have been particularly affected by the nutritional transition, overweight and obesity have become a challenge. In particular, Chile ranked seventh in adult obesity among the organization for economic cooperation and development members [3]. According to the Chilean National Health Survey 2009–2010, overweight and obesity reached 64.4% in men and 64.3% in women [4]. In the same period, the prevalence of diabetes mellitus was estimated at 8.4% in men and 10.4% in women [4]. Among children aged 6–18 years, overweight and obesity prevalence is estimated at 45.5% [5].

Although there are multiple risk factors associated to overweight and obesity, the literature shows that sugar sweetened beverages (SSB) are a significant risk factor for chronic diseases [6–9]. There is also evidence that intake of high energy dense and poorly nutritious processed and ultra-processed food is associated to obesity and metabolic syndrome [10, 11].

Chile is the second world’s largest consumer of caloric beverages, behind Mexico, with an estimated 180 l per capita consumption in 2014 [12]. In one decade, soda sales doubled and only 19.1% corresponded to calories-free soda [13]. Between 1987 and 2007, SSB consumption raised from 116 to 289 ml per capita per day and this increase was larger among poor households [14]. In the same period, in households from the metropolitan area of Santiago, household expenditures on soda, juices with added sugar and sparkling water -as a percent of total spending on food and beverages- increased from 4 to 8.4% [15].

Since the eighties, Chilean households have increased the proportion of expenditures in processed food with high energy density and added sugars and have reduced the consumption of traditional food. Percentage spending in processed food increased from 42 to 57%, and in the poorest households it went from 53 to 68% [14]. Consequently, processed and high-processed food represents 55.4% of all energy purchased by Chilean households in 2006–2007 [16]. Expenditures in food prepared outside the household increased from 12.1 to 20.1% between 1987 and 2007. Poor households show the largest increase [15].

Several fiscal policies to reduce the consumption of SSB have been recommended and implemented in different countries [17]. The World Health Organization (WHO) and the Pan-American Health Organization (PAHO) suggest implementing fiscal measures to discourage the consumption of foods and beverages that can harm health. For instance, the Plan of Action for the Prevention of Obesity in Children and Adolescents in the Americas, presented by the PAHO in 2014, advises taxing SSB and high-energy dense products in order to stop the increase in the prevalence of obesity in children and adolescents [18]. France, Hungary, Egypt, Finland, Mexico and the City of Berkeley in the USA have implemented taxes on SSB or high-energy dense foods. In Chile, there was a tax on sales and imports of beverages of 13% [13]. Since October 2014, the tax rate increased to 18% for non-alcoholic beverages, naturally or artificially flavored, that have content greater than 15 g of sugar per 240 ml or with an equivalent portion [19].

Despite the implementation of taxes in several countries, there is limited published scientific evidence on the effectiveness of fiscal measures in reducing the consumption except for Denmark [20] and Mexico [21]. In the absence of such evidence, price-elasticity estimates are a useful tool in achieving the objective of measuring the effect of a fiscal policy on consumption and simultaneously to forecast potential substitution effects. Some factors can modify the expected or estimated response to a tax such as the tax incidence, that is how much the tax is passed on to consumers, or increase in advertising campaigns or promotion of taxed products in stores. However, price elasticities provide useful estimates of the potential response to changes in prices.

There is a large variability in the SSB price elasticity estimates in the world. A systematic review on price-elasticity studies of non-alcoholic beverages and foods indicate that the price-elasticity ranges from −0.27 to −1.0 [22]. In general, SSB show larger absolute values of elasticities, that is, their demand is more sensitive to changes in price. A systematic review of studies performed in United States, France, Brazil and Mexico showed a mean price-elasticity of demand of SSB of −1.2 [23]. A recent study in Mexico estimated that the price elasticity of SSB was −1.16% and between −1.06 and −1.29 for soft drinks [24, 25] In Ecuador, the price-elasticity of SSB ranges between −1.17 and −1.33 depending on the socioeconomic group [26]. In contrast, studies on price-elasticity of high-energy dense foods are scarce. A study in Mexico found that the demand of this type of food is elastic [25]. The wide variability in the estimation of price elasticities is partially explained by proportion spent on SSB, the availability of substitutes for SSB in each country, the data used and the methods to derive elasticities.

The objective of this study is to estimate the price elasticity of demand for soft drinks, other SSB and high-energy dense foods in urban areas in Chile. Our paper adds to the existing literature the estimation of a linear approximate almost ideal demand system (LA/AIDS) and a sensitivity analysis using two additional models: a quadratic almost ideal demand system (QUAIDS) and one-equation two-part model used in the literature to derive price elasticities to test of the robustness of our findings. Results from the study could be used in the re-design and evaluation of current fiscal policies related to food and beverages consumption, and the potential reduction in the prevalence of overweight and obesity in Chile.

## Methods

### Data

We used the VII Family Budget Survey (FBS) collected between November 2011 and October 2012 by the National Institute of Statistics in Chile [27]. The FBS provides information on income and expenditures in urban households and is an important input to calculate the Consumer Price Index. The FBS has a probabilistic, bi-etapic and stratified design. It distinguishes between two zones: Gran Santiago (the Federal Capital City) and the rest of the regional capital cities. The FBS includes information on beverage and food and other household expenditures and socio-demographic variables. The sample size is of 10,527 households. We used the expansion factors in order to take into account the survey design in our estimates.

### Empirical model

We estimated a Linear Approximation of the Almost Ideal Demand System (LA/AIDS) by Deaton and Muellbauer [28] for beverages and foods. The LA/AIDS model is specified as follows:

$$ {w}_{hzi}={\alpha}_i+{\displaystyle \sum_{j=1}^i{\beta}_{i j} \log {p}_{zj}}+\gamma \log \left(\frac{E}{P}\right)+{\displaystyle \sum_{k=1}^k{\delta}_{i k}{\eta}_{hzk}+{u}_{j zi}} $$

Where *w*_*hzi*_ is the food or beverage expenditure share for food or beverage group *i* for household *h* living in zone *z*; *P*_*zi*_ is the unit value for food or beverage *j* at zone level estimated as the ratio between purchases in kilograms and expenditure on category *j*, where the *j-th* good is the composite numéraire that includes the unit value of other foods and beverages not considered in the demand system [29]; *E* is total household expenditure on beverages and food included in the system, *η* are variables at household level (education, sex and age of the head of the household, and equivalent adults), and log*P* is the Laspeyres price index, defined as follows [30]:

$$ \log {P}_j={\displaystyle \sum_{i=1}^{j-1}{\overline{w}}_i}* \log {p}_{mj} $$

Where *P* is the unit value of the *j-th* beverage or food category, $\overline{w}$ is the mean expenditure share in the category and *m* is the number of zones. Own and cross non-compensated price elasticities of the demand for the categories included in the system were calculated as follows:

$$ \varepsilon =-{\delta}_i+\frac{\widehat{\gamma}}{{\overline{w}}_j}-\frac{\widehat{\beta}}{{\overline{w}}_j}\overline{w} $$

Where *ε*_*j*_ is the price-elasticity of the food or beverage category, *δ* equals 1 if it is own price-elasticity and 0 if cross price-elasticity, ${\overline{w}}_j$ is the mean expenditure share of food or beverage, $\widehat{\gamma}$ is the estimated parameter of the log expenditure, $\widehat{\beta}$ is the estimated parameter associated to the unit value of the food or beverage category. In order to treat the censored nature of our response variable, we first modeled the probability of participation, that is the probability of positive consumption of each category by using a *probit* model and then calculated the Inverse Mills Ratio (IMR). Afterwards we included the resulting IMR of each category into the respective equation of the demand system [31]. The LA/AIDS model was estimated by Ordinary Least Squares equation by equation. We also estimated price-elasticities for soft drinks by income quintile.

In sensitivity analysis, we additionally estimated a quadratic almost ideal demand system (QUAIDS) that adds a quadratic expenditure term to model a non-linear association with expenditure share [32]. The IMR was not included in this model since this model does not allow incorporating different variables in each food and beverage equation. We also estimated a one-equation two-part model, in which we first modeled the probability of a positive consumption of soft drinks using a *probit* model and afterwards an Ordinary Least Square (OLS) regression with the number of equivalent adults, zone, education of the head of the household, age of the head of the household, age squared, income and number of children under 5 years as covariates and calculated the price elasticity of participation (EP) using this formula [33]:

$$ E P=\frac{\beta_i}{\sqrt{2\pi}} \exp \left(-\frac{1}{2}{\left({\beta}^{\prime}\overline{X}\right)}^2\right)\frac{1}{E\left( Y\left| X\right.\right)} $$

where X is the vector of independent variables, *β* is the vector of corresponding coefficients, E(Y|X) is the average value of the estimated probability and *β*_*i*_ is the coefficient related to unit values.

We then estimated the intensity price-elasticity for households with positive purchases, using an OLS Regression for the logarithm of the quantity consumed as a function of the logarithm of the unit value and a set of co-variables that included income and price indices for soft drinks and all other food and beverages categories. In this model, the intensity price-elasticity is the estimated coefficient of the logarithm of the unit value. Total price-elasticity was calculated by adding the participation and intensity price-elasticities [34].

### Variables

We defined eight beverages and food categories, that can be complement or substitutes for each other: (1) milk (milk and powdered milk); (2) coffee, including teas, infusions and mate tea; (3) plain water; (4) soft drinks; (5) other SSB that include powdered soda, sport drinks, isotonic water, juices, fruit pulp, and flavored water; (6) sweet snacks, containing cookies and other snacks; (7) sugar and honey (sugar, sweeteners and honey); and (8) desserts, including candies, desserts, chocolate, and chewing gum. We estimated beverage and food expenditure share summing expenditures in each category and dividing by total expenditure in the eight categories.

Unit values were calculated dividing expenditures in each category by total amount in kilograms. When the item was powdered, we rehydrated it in order to covert all amounts in kilograms, as indicated by Crovetto [14]. We averaged unit values by zone (Gran Santiago and the other regional capital cities) and input this average value when the household lacked to report it, that is when expenditure is zero in the category. Unit values were replaced by the mean plus 2.5 standard deviation when they exceeded the mean +−2.5 standard deviations. We calculated the adult equivalents as followed: a 5 years old or younger individual equals 0.77 equivalent adults (EA); 6 thru 12 years equals 0.8 EA; 13 thru 18 equals 0.74 EA and an individual of 19 years and older equals 1 EA [35].

We adjusted the models for EA of the household, education (last grade completed), sex and age of the head of the household. All models were estimated using STATA v. 13. To estimate the QUAIDS, we employed the Stata program provided by Poi [36].

## Results

Table 1 presents the proportion of households with positive purchases among the included categories in the demand system. Plain water shows the lowest percentage of households with positive expenditure. In contrast, 77.2% of Chilean households report spending in soft drinks. There is also a high prevalence of households with positive expenditures in sweet snacks (Table 1).

**Table 1:** Proportion of households with positive expenditures in beverages and foods. VII Family Budget Survey, 2011–2012^a^

Category	*n* with expenditure > 0	%	CI 95%
Milk	6225	59.5	(58.3, 60.8)
Coffee tea, mate and other infusions	4828	45.0	(43.7, 46.3)
Plain water	2496	23.0	(21.9, 24.1)
Soft drinks	8078	77.2	(76.1, 78.3)
Other flavored beverages	7004	66.5	(65.2, 67.7)
Sweet snacks	8297	78.0	(76.9, 79.1)
Sugar and honey	4562	43.2	(41.9, 44.4)
Desserts	6100	57.0	(55.7, 58.2)

^a^ Weighted using survey design (expansion factors) to represent populations in urban households

Table 2 shows the average unit values of beverages, snacks and desserts reported by the households with positive purchases, in 2011–2012 Chilean pesos. Beverages are cheaper by kilogram than snacks and desserts. Among beverages, soft drinks are more expensive than milk, coffee, tea and infusions, and plain water. Socio-demographic characteristics of the households show that in Gran Santiago 58% are headed by men and 62% in regional capitals. There is no difference in the age of the head of the household, or in household size across zones. However, in Gran Santiago the percentage of heads of the households with graduate level of education seems slightly higher than in the rest of regional capital cities (Table 3).

**Table 2:** Unit values (derived prices) of beverages, snacks and candies. VII Family Budget Survey, 2011–2012^a^

Category	Mean unit value	Minimum	Maximum	CI 95%
Milk	709.7	314.9	1836.0	(703.6, 715.7)
Coffee, tea, mate and other infusions	275.3	75.9	461.9	(273.6, 276.9)
Plain water	698.5	25.7	2347.1	(677.5, 719.6)
Soft drinks	766.6	47.9	2128.6	(755.7, 777.5)
Other flavored beverages	942.2	79.3	1978.3	(934.7, 949.6)
Sweet snacks	2546.7	860.9	4697.0	(2531.7, 2561.8)
Sugar and honey	1036.6	193.5	2708.9	(1025.5, 1047.8)
Desserts	5764.2	1786.9	10867.4	(5723.3, 5805.2)

^a^ Weighted using survey design (expansion factors) to represent populations in urban households

**Table 3:** Sociodemographic characteristics of households in Chile, by zone. VII Family Budget Survey 2011–2012^a^

Variable	Gran Santiago	CI 95%	Regional capitals	CI 95%	Total	CI 95%
Sex of head of the household (%)						
Male	57.7	(55.7, 59.6)	61.8	(60.4, 63.2)	59.4	(58.1, 60.7)
Female	42.3	(40.1, 44.3)	38.2	(36.8, 39.7)	40.6	(39.3, 41.9)
Age of head of the household (years)						
Mean	52.1	(51.5, 52.7)	52	(51.5, 52.5)	52.1	(51.7, 52.5)
Equivalent adults						
Mean	3.3	(3.2, 3.4)	3.2	(3.1, 3.2)	3.3	(3.2, 3.3)
Education level of head of the household (%)						
Kindergarden	0.6	(0.3, 1.4)	0.3	(0.2, 0.5)	0.5	(0.3, 0.9)
Elementary	8.4	(7.3, 9.6)	6	(5.3, 6.7)	7.4	(6.7, 8.1)
Secondary School	47.5	(45.5, 49.4)	43.6	(42.1, 45.1)	45.8	(44.6, 47.1)
Upper tertiary	38.2	(36.3, 40.1)	46.1	(44.6, 47.5)	41.5	(40.2, 42.8)
Post tertiary	5.3	(4.6, 6.2)	4.1	(3.6, 4.7)	4.8	(4.3, 5.4)

^a^ Weighted using survey design (expansion factors) to represent populations in urban households

Table 4 shows the results of the own and cross price elasticities with respect to soft drinks using LA/AIDS. The left half of the table shows the results of the models that include the IMR. The price-elasticity of all the eight categories is elastic. The price-elasticity of soft drinks is −1.37 implying that a 10% increase in price would be followed by a decrease of 13.7% in the amount consumed, which shows an elastic demand. The most extreme case is plain water, with a price-elasticity of −3.20. This implies that the demand of plain water is very sensitive to changes in price. The estimations of cross-price elasticities show that the degree of substitution of soft drinks with plain water is higher compared to other beverages and high-energy dense foods. A price increase in soft drinks is also associated with a higher quantity consumed of milk, coffee, tea and infusions, other sweetened beverages, sugar and desserts. On the right side of the table, the results of the model estimated without including the IMR. The estimates are robust, with no great differences with respect to the models that include the IMR.

**Table 4:** Own and cross non compensated price elasticities with LA/AIDS model. Chilean households. VII Family Budget Survey, 2011–2012

Category	Own price elasticity	Cross price elasticity with soft drinks	Own price elasticity	Cross price elasticity with soft drinks
Milk	−1.80* (0.07)	0.25 * (0.02)	−1.77* (0.07)	0.25* (0.02)
Coffee, tea, mate and other infusions	−1.62* (0.11)	0.10* (0.01)	−1.56* 0.11)	0.10* (0.01)
Plain water	−3.20* (0.08)	0.63* (0.01)	−3.20* (0.08)	0.62* (0.01)
Soft drinks	−1.37* (0.03)	--	−1.37* (0.03)	--
Other flavored beverages	−1.63* (0.06)	0.23* (0.02)	−1.68* (0.06)	0.23* (0.02)
Sweet snacks	−1.18* (0.04)	0.01* (0.02)	−1.18* (0.04)	0.0 (0.02)
Sugar and honey	−1.74* (0.12)	0.15* (0.01)	−1.75* (0.12)	0.15* (0.01)
Desserts	−1.12* (0.07)	0.03* (0.01)	−1.12* (0.07)	0.03+ (0.01)

* significant at *p* < 0.001, + significant at *p* < 0.1, standard error in parenthesis

**Fig. 1:**
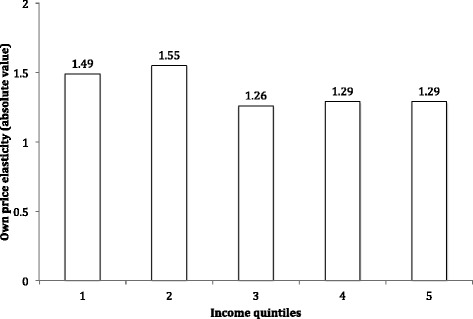
Price elasticity of the demand of soft drinks by income quintile. VII Family Budget Survey, 2011–2012

Figure 1 shows the price-elasticity of soft drinks by income quintile. As we can see, the population is fragmented into two major groups. Although all quintiles present an elastic demand to soft drinks, the first and second quintiles show a greater price-elasticity, whilst the third, fourth and fifth income quintiles are less sensitive to changes in prices.

Table 5 displays the results of the QUAIDS model. Unfortunately, it is not possible to get standard errors or *p*-values of price-elasticities using the QUAIDS Stata command. However, the results using this model are quite similar than those of the LA/AIDS model. The estimated price-elasticity for soft drinks is also −1.37 and the cross-price elasticities show the same sign and magnitude when compared to results from LA/AIDS model.

**Table 5:** Own and cross non compensated price elasticities using a Quadratic Almost Ideal Demand System model (QUAIDS). Chilean households. VII Family Budget Survey, 2011–2012

Category	Own price elasticity	Cross price elasticity with soft drinks
Milk	−1.78	0.25
Coffee, tea, mate and other infusions	−1.57	0.12
Plain water	−3.24	0.63
Soft drinks	−1.37	--
Other flavored beverages	−1.70	0.22
Sweet snacks	−1.21	0.00
Sugar and honey	−1.73	0.17
Desserts	−1.13	0.02

Lastly, we derived from the two-part model the price-elasticity of participation (−0.19) and the price-elasticity of intensity calculated on households with a positive consumption of soft drinks (−1.05). Hence, the total price-elasticity using this two-part model is of −1.24, slightly lower that the estimated using the LA/AIDS and QUAIDS.

## Discussion

We estimated a demand system for beverages and high-energy dense foods using a cross-sectional survey in Chile. We found that the demand for SSB in Chile is elastic (−1.37 for soft drinks and −1.67 for other SSB). Likewise, we provided evidence that an increase in soft drinks’ prices could lead to increases in the demand of other goods, such as plain water, milk, coffee, teas or other SSB. The sensitivity analysis shows very similar results, price-elasticity of soft drinks ranges from −1.24 to −1.37.

To our knowledge, this is the first paper that estimates price elasticities of the demand for soft drinks in Chile. Our findings are similar to other studies. Cabrera found an average price-elasticity of SSB of −1.29 in several countries [23]. Our estimate for soft drinks is also in the range of other Latin American countries, such as Mexico or Ecuador (−1.06 and −1.20, respectively) [24, 26]. The variability could be due to different definitions of SSB, different statistical models and also by the particular nature of demand in each country. Regarding income level, the studies by Colchero [24] and by Paraje [26] agree that population in lower income groups are more responsive to changes in price, as we found in the Chilean case.

Cross price-elasticities allow us to classify the rest of categories as substitutes and complements. Since their cross price-elasticity is positive, our study suggests that milk, coffee, tea, mate and other infusions, plain water, other SSB, sugar and honey and desserts show a substitute behavior towards soft drinks. For instance, regarding water, our preferred model shows that a 10% increase in price would be associated to an increase of 6.3% of plain water. However, own price-elasticity of water is high, perhaps because of the low proportion of households that report positive expenditures on this item and the high quality of private water supply systems in Chile. Cross price-elasticities of milk, tea, coffee, other infusions and other SSB are smaller, which means that substitution for these products is less clear than water’s.

We acknowledge several limitations in our study, which come primarily from the data. First, we were not able to split the category of soft drinks into non-diet or diet soft drinks, which would have been valuable in order to isolate the possible effect of a tax on soft drinks with added sugar from drinks without calories. Nevertheless, only around one fifth of soft drink consumption in Chile is on calories-free soda [13]. Since our data is cross-sectional, it is also likely that purchases of food and beverages included in our analysis are under-reported, because it excludes expenditures for consumption outside the household and does not consider purchases of all the households members.

Information on geographic location size, or a cluster variable to a more disaggregated level than zone was not available in the data set. Since the Family Budget Survey did not include information on prices that households face, we rather computed the unit values by dividing the amount of reported expenditure on the analyzed food and beverage category by the quantity purchased in kilograms. As noted by Deaton, this introduces two types of biases. First, there are measurement errors that come from both the numerator and denominator of the unit value calculation [37, 38]. Second, a quality effect is also present: households’ characteristics such as size or income affect unit values, because the products are also chosen by their quality. Deaton proposes a system of two equations for each category, which based on certain conditions, allows estimating price and budget elasticities while controlling for differences in the quality of the goods and measurement errors. In spite of its theoretical advantages, we were not able to estimate this Deaton’s model because the analyzed households must belong to small enough geographic units (such as villages) to support the assumption that the prices they face are the same. Deaton’s method assumes that variations in unit values in the same geographic unit are due to differences in the quality of goods. Therefore, to implement his method, information on small geographic location is crucial. We lacked this, and then tried to create artificial clusters based on income and the two zones available in the data set. Nonetheless, we got implausible price-elasticities estimates that we think were caused by the artificial nature of the clusters that we created and the small number of them. Furthermore, the FBS was collected along almost 1 year. Prices could have varied during this period. Regrettably, from the public microdata, it is not possible to know each household’s day of interview, so monetary variables are inflation adjusted. However, Chile presented a low inflation rate between 2011 and 2012 of 3.3 and 3.0%, respectively. Likewise, seasonality of purchases was not considered which could bias our price-elasticity estimations if the pattern of beverages consumption varies according to weather changes.

We also recognize the potential endogeneity due to omitted variables if the unit values are correlated with unobservable variables that influence demand. Ideally, we would have desired to at least aggregate unit values at a geographic level but the data set has only two broad zones. We could have also used predicted unit values from a first stage by calculating the expected unit value using ordinary least squares or generalized linear models adjusting for socio-demographic variables. Unfortunately, this imposed us a trade-off, since doing so introduced collinearity with the variables included in the demand system. Instead, in households without reported unit value we imputed using the averaged unit values at zone level and preferred to use a set of demographic variables to model the decision to purchase the items and so include the IMR into the equations of the demand system.

Despite these data limitations, the LA/AIDS with IMR is our preferred model for several reasons. First, it allows to test the conditions of homogeneity and symmetry through linear restrictions on fixed parameters [28]. The system also produces low standard errors when the number of commodities is greater than six [39]. It also allowed us to estimate cross price-elasticities with standard errors. In addition, the LA/AIDS results are somewhat similar than those produced by the QUAIDS and the two-part model. This gives us confidence that the price-elasticity that we estimated is robust.

## Conclusion

Our price-elasticity estimates provide essential information to policy design and evaluation. The evidence that we present here suggests that the demand for soft drinks is elastic in Chile. If the recent increase of 5% in the rate of taxes on naturally or artificially non-alcoholic beverages fully passes through prices, we would expect a decrease ~ 6.85% in the consumption of soft drinks and 8.15% in the consumption of other SSB, *ceteris paribus*. Simultaneously, an increase of 5% in the price of soft drinks would cause an increase about 3% in the consumption for plain water and to a lesser degree in the demand of other beverages, such as milk, coffee, tea and mate. These results however would depend on the type of tax and the pass-through prices.

The type of tax on sweetened beverages produces different outcomes. A specific tax (a fixed amount of money per physical unit of product) has several advantages. It is easier to administer and provides more stable fiscal revenue than *ad valorem* tax (a percentage of the value of the product) [40]. In addition, it reduces the gap between expensive and cheap brands [41]. The specific tax should at least be indexed to inflation to avoid that it dilutes over time. It is also feasible to implement a mixed tax system, where specific and *ad valorem* taxes coexist. *Ad valorem* taxes have the advantage that they are automatically adjusted for inflation. In any case, since a tax and its subsequent price increase implies a money transfer from consumers to government, careful assessment of health and economic impacts should be done. In one hand, it is possible that reduction in consumption produces health gains, through weight loss, reduced risk of metabolic syndrome and other desirable effects on health. These consequences are to be seen in the medium and long term and only after a significant increase in prices. Low taxes on sweetened beverages have little or no impact on body weight for instance [23, 42]. Substitution to other caloric beverages is also likely and the reduction of calories from soft drinks could be offset by increases of calories in the other products. Additional fiscal revenue should be returned to consumers to reduce potential regressivity effects of the tax, such as providing public drinking fountains, and education programs to reduce the information asymmetry.

## Abbreviations

EA: Equivalent adult
EP: Elasticity of participation
FBS: Family Budget Survey
IMR: Inverse Mills Ratio
LA/AIDS: Linear Approximation of al Almost Ideal Demand System
NCD: Non-Communicable Diseases
OLS: Ordinary Least Squares
PAHO: Pan American Health Organization
QUAIDS: Quadratic almost ideal demand system
SSB: Sugar sweetened beverages
USA: United States of America
WHO: World Health Organization
